# Whither the roads lead to? Estimating association between urbanization and primary healthcare service use with chinese prefecture-level data in 2014

**DOI:** 10.1371/journal.pone.0234081

**Published:** 2020-06-03

**Authors:** Sheng Nong, Zhuo Chen

**Affiliations:** 1 Youjiang Medical University for Nationalities, Baise, China; 2 Peking University, Beijing, China; 3 University of Georgia, Athens, GA, United States of America; 4 University of Nottingham Ningbo China, Ningbo, China; China Agricultural University, CHINA

## Abstract

With the rapid economic development across China over recent decades, examining how urbanization may affect healthcare service use and its implications is more than urgent. This study estimates the association between urbanization and primary healthcare services use in China. We construct a prefecture-level dataset on healthcare services utilization and urbanization. We regress the proportion of residents using healthcare services in primary healthcare centers versus secondary or tertiary hospitals on a set of prefecture-level control variables. Results suggest that use of primary healthcare centers outpatient service is positively associated with being in the proximity of a provincial capital, but negatively correlated with the percentage of the urban population and the availability of public transportation. Higher likelihood of seeking care in major hospitals instead of primary healthcare centers is associated with urbanization, justifying a need for primary care physicians as gatekeepers in China’s healthcare delivery system.

## 1. Introduction

Primary healthcare services are often provided by local community health centers for convenience and efficiency. However, at times patients may seek care at major hospitals away from where they live, even for conditions treatable at local community health centers–commonly known as bypassing. In countries where primary care physicians serve as gatekeepers, bypassing is less likely to be an issue for their healthcare systems. However, patients in China have more latitude in choosing a provider within their home province than most other healthcare systems thus bypassing is more common in China. In addition, major Chinese hospitals are motivated to compete for patients, leading to wide-spread bypassing and a significant economic burden and inefficiencies in the healthcare system. Bypassing causes congestion in major hospitals and underutilized resources in lower-tiered providers, resulting in wasted resources, low patient satisfaction, and medical malpractice disputes [1].

Prior research argued that primary care-centered healthcare delivery systems are more efficient than hospital-centered systems [2]. Patient flow managed by primary care physicians plays a crucial role in proper patient management and reducing patients’ search costs in accessing specialty care services [3]. Earlier studies provide ample support for the impact of gatekeeping in reducing inefficiencies and trimming costs [4–6]. With this backdrop, reducing bypassing in the healthcare delivery system has been one of the focal areas of China’s health reform since 1997. In a recent national policy, China’s central government pushed for gatekeeping by using the phrase ‘Fen Ji Zhen Liao’ (i.e., two-tier healthcare) to describe the process of ‘patients visiting a primary healthcare center (PHC) as a point of entry into the healthcare systems and followed by a two-way referral’ [7]. This is commonly known as gatekeeping, with a referral after the first point of contact service [1,8,9].

### China’s healthcare system and referral practices

From 1949 to 1970s, China had a tiered healthcare system operating under the urban-rural dual social structure and the planned economy. Healthcare was provided through village clinics, township health centers, county hospitals, and city hospitals to rural residents, and employer-sponsored clinics, community health centers, district and city hospitals in urban areas. Community clinics, health centers, rural village clinics, and township health centers are considered PHCs in China. Before 1978, Chinese residents’ mobility was restricted by their residential registration (known as hukou) and employment. Patients could receive reimbursement if they followed the referral policies set in place by their employers or villages. A national policy promulgated in 1978, *‘Rectifying and Enhancing Free Medical Services’*, banned reimbursement for medical expenses without documentation of proper referral or pre-approval from a physician.

In 1978, China launched a national economic reform from a planned economy to a market economy, with no exception for the healthcare sector. Higher utilization of healthcare services has accompanied stronger economic growth. However, bypassing was rare during the 1980s when most PHCs could treat common health conditions. To address the imbalance between the strong demand for quality health services and a shortage in the supply of health services, the Chinese government promulgated market-oriented health policies to improve health financing [10]. The central government has issued policies encouraging hospitals to broaden their sources of revenue, expanding hospitals’ autonomy, and permitting hospitals to use their surplus funds [11–13]. During this period, the quantity and size of public hospitals were included in the performance evaluation of local governments, and therefore healthcare delivery systems experienced a substantial expansion [14]. In 2000, China’s then Ministry of Health enacted a policy to allow patients to choose a physician when seeking healthcare [15], which officially ended the practice of mandated referral.

On the financing side, China’s social medical insurance schemes have relaxed the traditional referral rules. China established the national Urban Employee Basic Medical Insurance (UEBMI) to cover the medical needs of all formal urban employees in 1998. The funds in a UEBMI personal account are owned by the enrollee and can be spent at any level of delivery system without referral permission. The funds in the pooled account follow the ‘first-come-first-serve’ rule, and can be used in any contracted hospitals and pharmacies [16]. Two other social insurance schemes, namely the New Rural Cooperative Medical Scheme (NRCMS) and the Urban Resident Basic Medical Insurance (URBMI) that were established in 2003 and 2007 respectively, have followed UEBMI in their policies regulating the reimbursement procedure, to allow beneficiaries to choose providers at any levels [16].

Given China’s economic development and misaligned incentive mechanism for the providers, small PHCs with limited facilities and inadequate capacity tend to lose in the competition for patients and form a feedback circuit of losing medical staff and then patients again. Even with a tiered reimbursement rate in favor of PHCs, the price effect does not offer enough incentive to attract patients to use PHCs. Bypassing thus has become common.

China’s healthcare delivery system faces the challenges of the more than 20 years of social norm among patients of utilizing hospital as primary source of healthcare services, shortage of a capable and motivated primary care workforce. To rebuild the referral system, China’s healthcare system has focused on supply side efforts. Since 1997, China has issued at least 60 policies aimed to address bypassing in its healthcare delivery system. Measures implemented include investing in PHC infrastructure and medical equipment, providing training and assistance to improve PHC capacity, and subsidizing medical students from rural areas to work with PHCs in targeted areas [1,7,17,18]. The policy objectives of these measures are to enhance PHC capability to treat patients, thus to reduce bypassing. However, the impact of these demand side efforts has not been systematically assessed elsewhere.

### Urbanization and bypassing

Recent studies on bypassing have taken two different perspectives, demand side and supply side. Demand side research focuses on the determinants of patient choices [17,19] and the supply side concerns on how the physician services and payment schemes drive the patient flow [8,20,21]. Prior studies on this topic often collected individual-level data from face-to-face surveys and assumed a static demographic and location profile. However, because individual-level studies are often limited in sample size thus relevant to regional settings, few studies have examined the macro-level determinants including urbanization, one of the most remarkable changes occurred in China within the last 40 years. From 1979 to 2016, China’s urban population has increased annually by 4.1%, while its rural population decreased by 0.8% each year on average. In 2016, the migrant population in China totaled 254 million, which is more than the population of England, Germany, France, and Greece combined [22]. Urbanization affects human health [23], in areas such as non-communicable diseases [24] including cancer [25], aortic stiffness [26], kidney stones [27], obesity [28], mental health [29,30], infectious diseases [31,32,33], and PM 2.5 related mortality [34]. Studies also show an association of urbanization with health services utilization such as hospital admissions for alcohol and drug abuse[35] and liver transplant [36]. Other studies have emphasized the impact of urbanization on health disparity [37] and income inequality [38]. How urbanization relates to health services utilization across different tiers of healthcare is a critical question requiring attention and research.

Urbanization increases the proportion of people living in urban areas and changes the ways how society adapts to economic transition [39]. With the population movement from villages to cities intensifies, urbanization may lead to reduced utilization of rural PHC. Urbanization is often seen as a double-edged sword to rural health services utilization. While urbanization improves the service capability of PHC thus has a ‘pull-back effect’ from high-tier healthcare providers, it also has a ‘consumption upgrade effect’ which leads to more intensive bypassing [40]. From 1978 to 2017, the percentage of non-agricultural labor in China increased from 30.2% to 73.0%. The disposable income reached RMB 33,616 Yuan per person (about $4,831 at an exchange rate of $1 = RMB 6.96 as of July 23, 2019) among urban population and RMB 12,363 Yuan per person ($1,776) in rural areas, both of which are about ten times more than those in 1978. The proportion of vehicle ownership in China increased by 13.8% and the social medical insurance savings account rose by 20.7% each year. With these rapid changes, population mobility due to urbanization have a profound impact on health services utilization.

**Fig 1:**
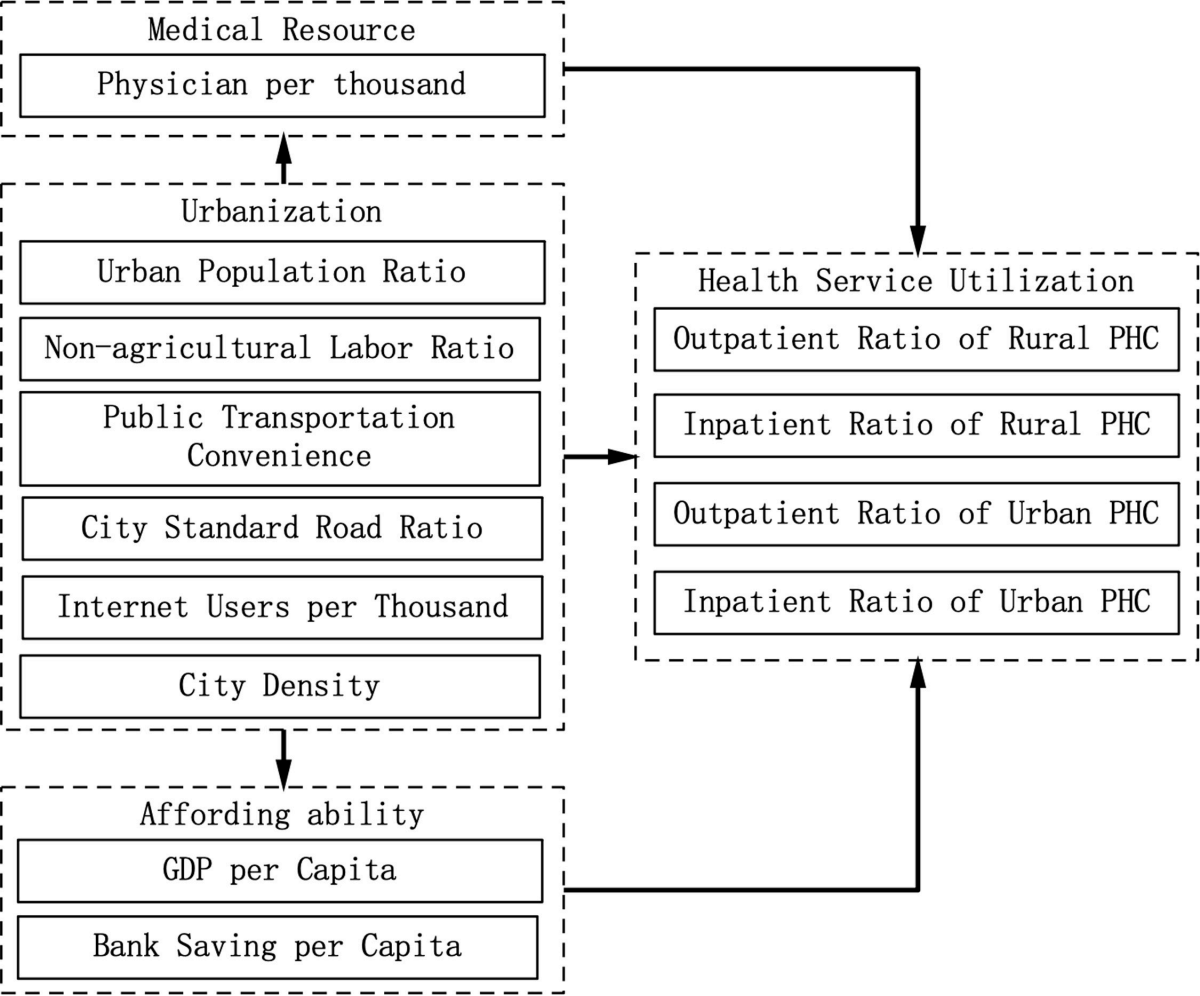
Urbanization and health services utilization.

Fig 1 illustrates how urbanization can affect health services utilization on both supply and demand sides. On the demand side, the adoption of innovative agricultural technologies has increased production efficiency and freed a great deal of labor from agriculture. In China, migrant workers on average earn more than farmers, so their ability to afford services at high-tier providers has improved, leading to an income effect [39]. Besides a reduced share of the agricultural population and increasing urban population density, urbanization also causes improvements in transportation infrastructure and additional internet users. The demand side changes may cause the redistribution of the medical workforce.

Urbanization affects bypassing in combination with other factors. The *first* factor we consider is that insurance policies have eased migrant workers’ movement and employment but has an unintended incentive for bypassing. China has four main insurance plans for its citizens, namely UEBMI for urban formal workers, NRCMS for rural residents, URBMI for urban residents with informal jobs or no jobs, and Social Medical Aid (SMA) for eligible low-income individuals. Migrant workers are mostly covered by NRCMS and URBMI. If a migrant worker wants to bypass PHCs and visit secondary or tertiary providers directly, they can inform the insurance administrative department by phone and submit the required documents after their visit. The reimbursement rate is the same, whether they have used a referral or not. Although this was designed to ensure migrant workers having adequate access to health services, it has an unintended consequence of increased bypassing. In addition, one investigation showed that migrant workers in China consider a referral from PHC physician burdensome, because, if a PHC is unable to treat their condition, they will have to find a specialist and pay extra fees [41]. Personal level of referral service is not required and often considered a luxury among Chinese patients.

The *second* factor that has facilitated bypassing is the reduced cost of transportation. The development of the bullet train and public transportation system in urban areas has drawn patients from the rural area, bypassing local PHC in the rural areas. Because of their high wage rates and thus high opportunity cost, migrant workers living in urban areas usually do not intend to spend additional time to travel back to their hometown for PHC services, in which case a higher reimbursement rate may apply. They also consider urban major hospitals more competent than rural hospitals; thus the absolute number of rural PHC utilization may decrease. In the urban areas, those who are employed may visit an urgent care unit of major hospitals when they are off work. A recent study showed that Chinese urban residents have two modes of health services use [42]. When they fell sick, they tend to self-diagnose or rely on informal sectors. If the symptoms are not relieved, they bypass PHCs and visit major hospitals in part to save time and transportation costs.

The *third* factor relates to the financing of China’s PHCs. The Chinese government subsidizes PHCs following the ‘Separating Revenue and Expenditure’ (shouzhi fenkai) policy, through which PHCs transfer all revenues at the end of the year to upper-level management and in turn receive a fixed amount of budgeted funding. If one PHC secures more revenue than expected, it will be subsidizing other PHCs in the same tax district. The policy has been designed and promulgated to reduce “induced demand” but led to misaligned incentives as an unintended consequence. Many PHC workers have left for better-paid jobs, and those who stay have little incentive to provide more or better quality of services, as their workload is not related to their income. For ten years after this policy was implemented, PHCs have seen an average reduction of 10% in hospital beds and clinical services [1]. However, high tier hospitals are free from this policy and can reward their workers with a revenue surplus; thus they have an incentive to attract patients.

This paper intends to examine the association between urbanization and health services utilization using prefecture-level data in China in 2014. This research is to understand bypassing in the backdrop of rapid urbanization and to examine how aspects of urbanization, including population structure and transportation, affect health services utilization. The study fills a critical knowledge gap in understanding how urbanization is related to health services utilization in rural and urban settings by using an innovative dataset linking health services utilization and prefecture-level indicators. The results of our study will have important implications for formulating policies to reduce bypassing. We describe the data and methods below, then present the results and discussions, and end with our conclusions.

## 2. Materials and methods

### Data and variables

Health services utilization data are from *the National Health Financial Yearbook of 2014*. China’s public hospitals submit annually a set of statutory data to the local health administrative department through an online system. The data include hospital income and expenditure statements, balance sheets, and service provision portfolios. The data are collected and reported from county health bureaus to prefecture-level authorities, and then to provincial and national-level health authorities.

To link health services utilization with city-level indicators, we use prefecture as the unit of study. Prefecture-level information comes from the *China City Statistics Yearbook of 2015* [43], including population, economic, and geographic data in 2014. Cities in Xinjiang and Tibet are excluded due to their sparsely populated geography. We group prefecture-level districts in a province-level metropolitan city (i.e., Beijing, Shanghai, Tianjin, and Chongqing) as one because the distance within the cities is not an issue for patients to seek care outside of their residential districts. The *National Health Financial Yearbook* does not record China’s privately-operated hospitals, but we believe that the data from public hospitals are representative at least up until 2014. First, most Chinese citizens trust and use public hospitals, which provide more than 90% of outpatient and hospitalization services in the country [44]. Second, the data for health services utilization were extracted directly from each hospital’s management information system, ensuring its quality. We are unable to obtain private providers’ utilization data at the prefecture-level. By using the year 2014, we avoid the impact of the national policy enacted in 2015 which aimed to increase the health services utilization at PHCs [45], a topic for future researches. Our final dataset is a cross-section of 274 records of Chinese cities in 2014.

The dependent variables are the four variables listed on top of Table 1.They represent the outpatient and inpatient utilization in rural or urban PHCs, which is equal to the annual outpatient (inpatient) service volume of urban or rural PHCs divided by total annual rural or urban outpatient (inpatient) service of the city. The other variables are as follows. *Capital* is a dummy variable of city location, taking the value of one if a city is the provincial capital city or bordering the capital city, and otherwise zero. The percentage of the urban population (*Urban Population*) reflects the extent of urbanization in the administrative areas of the prefecture; it is the most used urbanization index in previous studies [24,30,35,40,46]. The ratio of non-agricultural labor (*Non-Agricultural Labor*) reflects how many residents were working in non-agricultural industries, including both manufacturing and service, and thus revealing the economic structure of the prefecture.

**Table 1:** Descriptive statistics of prefecture-level data in China, 2014.

Variable	Unit	Description	Mean	SD	Min	Max
***Dependent variables***						
*Urban Outpatient*	%	Percentage of urban PHC outpatient service (among all urban outpatient service)	6.06	7.35	0.00	55.04
*Urban Inpatient*	%	Percentage of urban PHC inpatient service (among all urban inpatient service)	1.33	1.63	0.00	7.98
*Rural Outpatient*	%	Percentage of rural PHC outpatient service (among all rural outpatient service)	35.03	13.14	0.00	66.88
*Rural Inpatient*	%	Percentage of rural PHC inpatient service (among all rural inpatient service)	22.92	12.91	0.00	54.44
***Independent variables***						
*Urban Population*	%	Percentage of urban population in the prefecture	34.88	22.55	4.83	100.00
*Non-Agricultural Labor*	%	Percentage of non-agricultural labor	87.51	7.71	51.93	99.48
*Population*	10,000 persons	Total resident population of the prefecture	445.01	318.38	24.10	3366.80
*Urban Density*	Persons per km^2^	Urban population divided by urban area	879.45	729.89	14.91	4271.13
*GDP*	RMB per capita	Per capita GDP of the prefecture	48603.11	28076.32	10171.00	200152.00
*Doctor*	Persons per 1000	Number of public and private doctors per thousand persons in the prefecture	2.17	0.96	0.79	7.30
*Savings*	RMB per capita	Average bank saving per capita	33737.01	23723.63	10577.39	180936.00
*Internet*	Users per 1000	Number of internet users per thousand persons	177.06	142.74	29.33	1000.00
*Bus*	Per 10,000	Number of bus per 10 thousand persons	7.92	5.15	0.59	31.43
*Road*	m^2^	Road area in meter squared per capita in the prefecture	12.97	8.61	1.17	106.27

Total number of cities: 274.

The population of the prefecture or metropolitan city (*Population*) is included as a control variable. The population density of the urban area (*Urban Density*) is equal to the number of urban residents divided by urban area in square kilometers. GDP per capita (*GDP*) is used to capture the level of economic development of the prefecture. The number of doctors per thousand population in the prefecture (*Doctor*), including those in the public and private sectors, is a proxy of the healthcare workforce in the city. We were unable to find data relating to the income level of each prefecture, so the resident average bank saving (*Savings*) was used as a proxy of the wealth of the average resident in the prefecture. Chinese people have the tradition of saving for catastrophic events such as major illnesses. Bank savings is correlated with the propensity and ability to use high-quality healthcare services. Internet users per thousand population (*Internet*) correlates with the accessibility of information. Residents tend to find it easier to obtain information about their health and their providers if internet use is more accessible. The number of public buses per ten thousand (*Bus*) is a proxy of convenience and cost of transportation. The city road area in square meters per capita (*Road*) reflects the urban public transportation capability, with higher numbers indicating more public transportation facilities and more vehicles in the prefecture.

### Empirical model

Consider the following model (1),

$$y_{ij}=\beta_{i0}+\sum \beta_{ik}*x_{ikj}+\mu_{ij}$$ (1)

Where *y*_*ij*_ represents the dependent variable *i* of a prefecture *j*, *i* represents one of the four dependent variables; *x*_*ikj*_ is the independent variable *k* of prefecture *j* for dependent variable *i*, *k* is the index of the independent variables; *β*_*i0*_ is the intercept term, *β*_*ik*_ is the parameter of each *x*_*k*_ and *μ*_*ij*_ is the disturbance term of prefecture *j* for the dependent variable *i*. Dependent variables, in our estimation, are the annual percentages of PHC services utilization as we have explained previously. Patients may choose a PHC outpatient and inpatient service because of preference, distance, and provider reputation. If these unobserved factors affect the utilization of outpatient and inpatient services simultaneously, then the residuals are correlated. We use the Seemingly Unrelated Regression (SUR) estimation to address this concern [47].

Because China’s urban and rural areas differ in many important aspects and that urbanization is the main factor we intend to examine, separate regressions of rural and urban PHC healthcare service utilization are warranted. SUR was estimated for two groups of dependent variables separately, i.e., the urban group (*Urban Outpatient* and *Urban Inpatient*) and the rural group (*Rural Outpatient* and *Rural Inpatient*). Results of OLS regression are listed for comparison.

## 3. Results

Table 1 presents descriptive statistics. In general, urban residents use PHC health services less frequently. Rural residents have much higher usage than their urban counterparts, with 35% of outpatient services being within rural PHC facilities and 23% of inpatient services. About 35% of the prefectural population are urban residents, while about 87.5% of the labor force work outside the agricultural industry. The average prefectural population is 4.45 million with significant variations across the prefectures. The average GDP per capita is about RMB 48,603 Yuan, and their per person savings is RMB 33,737 Yuan. The number of buses per ten thousand residents averages at 7.92 and per capita, and the area of road is about 12.97 square meters. Internet users per thousand averages at 177 persons.

The regression results are in Tables 2 and 3. The location factor (*Capital*) is positive and statistically significant in the regression of rural PHC outpatient services utilization. The coefficient of the percentage of urban population (*Urban Population*) is negative and statistically significant in the equations for rural PHC outpatient services but positive in the regressions of urban PHC outpatient and inpatient services. The total city population (*Population*) is positively correlated to PHC rural outpatient services and urban PHC inpatient and outpatient services utilization. In contrast, the urban population density of the prefecture (*Urban Density*) is not statistically significant in any of the regression equations, implying the population movement’s related with healthcare utilization.

**Table 2:** Determinants of rural PHC outpatient and inpatient services utilization: Prefecture-level data in China, 2014.

Dependent Var.	Rural PHC Outpatient Services		Rural PHC Inpatient Services	
	OLS	SUR	OLS	SUR
*Capital*	2.990*	2.940**	0.686	0.657
	(1.330)	(1.290)	(1.330)	(1.300)
*Urban Population*	−0.123**	−0.131**	−0.034	−0.036
	(0.039)	(0.038)	(0.040)	(0.039)
*Non-Agricultural Labor*	4.110*	4.350*	3.200*	3.240*
	(1.280)	(1.250)	(1.260)	(1.230)
*(Non-Agricultural Labor)*^*2*^	−2.370**	−2.530**	−0.020**	−0.020**
	(0.008)	(0.008)	(0.008)	(0.008)
*Population*	0.003	0.003	0.009**	0.009**
	(0.002)	(0.002)	(0.002)	(0.002)
*Urban Density*	−0.000	−0.000	0.000	0.000
	(0.000)	(0.000)	(0.000)	(0.000)
*Log*. *GDP*	−67.300	−67.500	−90.400*	−87.500*
	(39.700)	(38.700)	(41.400)	(40.300)
*(Log*. *GDP)*^*2*^	2.810	2.790	4.220*	4.080*
	(1.870)	(1.830)	(1.960)	(1.900)
*Doctor*	1.120	0.811	−0.555	−0.657
	(1.060)	(1.030)	(1.080)	(1.040)
*Log*. *(Savings)*	−5.200*	−3.030	−0.015**	−0.014**
	(2.560)	(2.410)	(0.006)	(0.005)
*Internet*	0.000	−0.000	−0.005	−0.007
	(0.006)	(0.006)	(0.006)	(0.006)
*Bus*	−0.437**	−0.462**	−0.387*	−0.394*
	(0.160)	(0.156)	(0.160)	(0.156)
*Road*	0.023	0.023	−0.133	−0.131
	(0.089)	(0.0867)	(0.090)	(0.087)
*Constant*	3.126	2.872	3.882	3.716
	(2.138)	(2.081)	(2.217)	(2.154)
*R*^*2*^	0.461	0.460	0.461	0.460

(1) N = 274; (2) Standard errors in parentheses; (3)

* p<0.05,

** p<0.01; (4) PHC: Primary Healthcare Center.

**Table 3:** Determinants of Urban PHC outpatient and inpatient services utilization: Prefecture-level data in China, 2014.

Dependent Var.	Urban PHC Outpatient Services		Urban PHC Inpatient Services	
	OLS	SUR	OLS	SUR
*Capital*	−0.098	−0.090	−0.036	−0.019
	(0.759)	(0.739)	(0.209)	(0.203)
*Urban Population*	0.083**	0.085**	0.017**	0.018**
	(0.023)	(0.000)	(0.006)	(0.006)
*Non-Agricultural Labor*	−0.828	−0.871	−0.062	−0.088
	(0.733)	(0.711)	(0.198)	(0.192)
*(Non-Agricultural Labor)*^*2*^	0.005	0.005	0.0004	0.0005
	(0.005)	(0.005)	(0.001)	(0.001)
^*Population*^	0.005**	0.005**	0.0009**	0.001**
	(0.001)	(0.001)	(0.000)	(0.000)
*Urban Density*	0.000	0.000	0.000	0.000
	(0.000)	(0.000)	(0.000)	(0.000)
*Log*. *GDP*	−75.000**	−75.000**	−15.800*	−17.600**
	(22.700)	(22.200)	(6.480)	(6.280)
*(Log*. *GDP)*^*2*^	3.680**	3.680**	0.778*	0.864**
	(1.070)	(1.040)	(0.306)	(0.297)
*Doctor*	0.404	0.457	−0.048	0.014
	(0.608)	(0.587)	(0.168)	(0.162)
*Log*. *(Savings)*	1.300	0.921	−0.000	−0.000
	(1.470)	(1.330)	(0.000)	(0.000)
*Internet*	0.405	0.426	−0.187*	−0.160
	(0.335)	(0.325)	(0.093)	(0.090)
*Bus*	0.039	0.043	−0.985	−0.586
	(0.092)	(0.089)	(2.510)	(2.440)
*Road*	−0.108*	−0.108*	−0.020	−0.021
	(0.0509)	(0.0496)	(0.014)	(0.014)
*Constant*	4.056**	4.100**	0.835*	0.936**
	(1.224)	(1.190)	(0.347)	(0.336)
*R*^*2*^	0.436	0.436	0.436	0.436

(1) N = 274; (2) Standard errors in parentheses; (3)

* p<0.05,

** p<0.01 (4) PHC: Primary Healthcare Center.

The percentage of non-agricultural labor (*Non-Agricultural Labor*) is statistically significant and positively related to rural residents’ use of PHC services. The direction of the quadratic term for *Non-Agriculture Labor* is inverse to the level term, which implies a quadratic effect for rural PHC services.

The natural log of per capita GDP (*GDP*) is negative and statistically significant in the regression of rural and urban residents’ PHC inpatient service utilization and urban PHC outpatient service utilization. Its quadratic term is positive in the equations for both rural and urban residents’ PHC inpatient services, and the effect for urban outpatient and inpatient services is statistically significant, indicating the concave function of the income effect for PHC utilizations.

The resident bank savings (*Savings*) is negatively associated with rural PHC inpatient service utilization but not with any other dependent variables, implying a negative income elasticity. The coefficient of the indicator for internet users (*Internet*) is negative in the regression for urban PHC inpatient service utilization, showing the impact of information accessibility toward PHC utilization. The coefficient for the number of buses (*Bus*) is negative and statistically significant in the equation for rural PHC outpatient and inpatient service utilization, which verifies our hypothesis that convenience of transportation will reduce resident’s moving cost of bypassing. The road coverage per capital (*Road*) is also negative and statistically significant in the regression of urban PHC outpatient service.

We examined the null hypothesis of the coefficients of *Urban Population*, prefecture *Population*, and *Urban Density* in each regression to test the effect of demographic and geographic features of a prefecture. Regression results imply that we cannot accept the null hypothesis at the significance level of 0.05.

## 4. Discussion

The results of this study suggest an important role of urbanization in residents’ decision to use PHC health services. Geographic location, public transportation, economic development, and internet usage are all correlated with the use of PHC inpatient or outpatient services.

A quadratic curvature of *Non-Agricultural Labor* in the regressions for rural areas indicate that, when the percentage of non-agricultural labor of a city increases, rural residents are more likely to use PHC services than major hospitals at first, but when the percentage increases to 85.3% and 81.4%, the rate of PHC service utilization starts to decline. Considering the average level of non-agricultural of 87% in 2014 and its increasing trend, we predict a declining trend in PHC service utilization, which is consistent with the fact that China’s PHC ratio (with rural and urban PHC ratio aggregated) is about 65% in 2009 but less than 35% in 2014 [48].

Our regressions have a somewhat surprising result–if a prefecture sits geographically next to a provincial capital city, its rural residents are more likely to use the local PHC outpatient services. This result has the appearance of contradicting our hypothesis that cities near provincial capitals lose patients because of the presumed syphon effect of major hospitals in the provincial capital city. However, we have compared the mean of inpatient ratio of cities bordering the provincial capital to that of the other cities using *t*-tests and found that the other cities have significantly higher rates of hospital service utilization than those of the cities bordering the provincial capital. This suggests that patients near capital cities are more likely to go to the capital city for healthcare services. We have two explanations for this finding. First, prefectures near provincial capitals tend to be wealthier than prefectures that far away from the capital, thus rural PHCs in these prefectures may be more competent in retaining local patients. Second, residents in these prefectures are attracted to providers in the provincial capital; thus they bypass the local secondary and tertiary hospitals as well and visit the major hospitals in the provincial capital directly. The latter scenario is consistent with the high rates of overall health services utilization and the PHC outpatient services use in the near-capital cities we have seen.

Industrialization offers rural residents with stable employment and incomes. Our results on GDP per capita suggest that this is positively associated with rural PHC services utilization. We examined several industrialized cities including Dongguan, Yiwu, and Kunshan, and found that most of the rural PHCs in these cities were established to provide health services to manufacturing plants nearby and thus increases the PHC services utilization. GDP per capita had a quadratic effect on PHC services used. A possible explanation is that PHCs in cities with a growing GDP would have improved capacity, thus the quality of PHC offsets the income effect of urban residents.

Rural residents’ choice among providers of different levels is sensitive to their wealth. Bypassing is more likely to occur if residents can afford high quality medical services in major urban hospitals. Another explanation is that a high income facilitates rural residents to migrate to urban areas, so the utilization of rural PHC declines with the increasing per capita saving.

The number of internet users was used to capture the effect of information and the cost of finding a proper provider. Many tertiary hospitals in China offer internet appointments for residents to improve accessibility, and this may explain why urban PHC inpatient services utilization is affected negatively by internet users, because larger hospitals have advantages in setting up an online presence.

Urbanization affects resident choices of healthcare services providers. We anticipate that when more population move into urban areas in the near future, rural and urban PHCs at the prefecture-level cities of China will face additional challenges.

## Strengths and limitations

This study is one of the first attempts to use city-level data to examine the impact of urbanization, among other factors, on the pattern of PHC health service utilization. By including an indicator of proximity to the provincial capital, we observed the siphon effect of provincial capital cities on health services utilization at secondary and tertiary hospitals in cities near the provincial capitals. However, this study has at least two limitations. First, we used an aggregate-level dataset, and therefore the ecological fallacy applies if we make inferences at the individual level. We refrained from doing so and our conclusions are applicable to the prefecture-level percentages and rates and may be relevant for policymaking at the prefecture-level (or city level). Second, we are unable to examine the impact of more recent policies, which are relegated to future investigations when the required data are available to the public. Nonetheless, our results offer important implications for policies governing health services utilization and/or urbanization.

## 5. Conclusions

This study draws three major conclusions. Firstly, geographic location and changes in the percentage of the urban population significantly affect bypassing in rural areas. As China will experience further urbanization with more physical and human capital flowing into urban centers, health services in rural areas may not be able to keep up with the demand among rural residents for high-quality medical services requiring specialized equipment. Bypassing arises not only from the gaps between the capacity of PHCs and that of higher-level hospitals, but also from such gaps between rural and urban areas, and between near-capital areas and other areas. Future policymaking efforts and additional investment into rural PHCs need to take into consideration urbanization and associated population mobility.

Secondly, residents’ wealth and prefecture-level transportation are associated with reduced PHC utilization, and this may indicate that recent tiered healthcare (‘Fen Ji Zhen Liao’) policies to reduce bypassing are offset by the macroeconomic factors. Inadequate gatekeeping, competition between hospitals, and an increase in the number of wealthy residents contribute to the rise in bypassing, exacerbating PHCs’ financial operations and medical capabilities.

Third, information flow encourages bypassing when the gap in the quality of health services provided by PHCs and hospitals continue to exist. Our analysis found that the number of internet users is associated with a lower probability of using urban PHC inpatient services because residents usually go to hospitals that can offer them convenient online appointments and payment facilities. Investing in telemedicine and health information infrastructure may improve the PHC capacity thus should be assessed.

To summarize, urbanization has a complex effect on the utilization of healthcare services. Whether the roads pave the way to enhanced PHC capacity for serving their constituents or lead them to urban centers for healthcare services will depend on the location, economic development, and rurality. More in-depth research is needed to examine the consequences of urbanization on China’s healthcare systems.

## Supporting information

S1 Data (ZIP)

## Abbreviations

PHC: Primary Healthcare Center
UEBMI: Urban Employee Basic Medical Insurance
NRCMS: New Rural Cooperative Medical Scheme
URBMI: Urban Resident Basic Medical Insurance
OLS: Ordinary Least Squares
SUR: Seemingly Unrelated Regression
