# Supplementary material for: Whither the roads lead to? Estimating association between urbanization and primary healthcare service use with chinese prefecture-level data in 2014
Source: PLoS One. 2020 Jun 3;15(6):e0234081. doi: 10.1371/journal.pone.0234081 (PMC7269333; doi:10.1371/journal.pone.0234081)
Supplement: S1 Data — (ZIP) [file pone.0234081.s001.zip › Nong_Chen_data_description.docx]

This data set records the medical utilization and city feature of China’s prefecture-level cities in 2014.

1. The medical utilization data in this research is variable 4,5,6,7,8,9, they came from *National Health Financial Yearbook of 2014*. Every year China’s public hospital has to summit this statutory report to local health administrative department through an online system. The yearbook records every hospital’s income and expenditure statement, balance sheet and medical portfolio information. The data was collected and reported from county to prefecture city, and then to provincial and national level. The *National Health Financial Yearbook* does not record China’s privately operated medical organizations

2. In order to match medical utilization to status of the city, we used prefecture city as sampling unit. In this data set they are variable 1-3, and 10-19. These city’s information came from *China City Statistics Yearbook of 2015*[1], it recorded population, economical and geographical data of Chinese prefecture-level cities in 2014. All the cities in Xin Jiang and Tibet province were excluded.

| **No.** | **Variable name in stata** | **Meaning** |
| --- | --- | --- |
| **1** | **City** | Prefecture city name |
| **2** | **Province** | The province of the city |
| **3** | **Nearcapital** | =1 means if the city is the provincial or border on provincial city |
| **4** | **phcoutratio (%)** | ratio of outpatient service of both rural and urban area |
| **5** | **phcinratio (%)** | ratio of inpatient service of both rural and urban PHC |
| **6** | **Ctyoutratio(%)** | ratio of urban PHC outpatient service |
| **7** | **Ctyinratio(%)** | ratio of urban PHC inpatient service |
| **8** | **Ruraloutratio(%)** | ratio of rural PHC outpatient service |
| **9** | **Ruralinratio(%)** | ratio of rural PHC inpatient service |
| **10** | **Urbanratio(%)** | urban area population/city population |
| **11** | **Sethirdpeo (%)** | non-agricultural labor/city total labor |
| **12** | **Population (10 thousand)** | total long stayed population of the city |
| **13** | **Ctydense (people per sq.km.)** | urban population/urban area |
| **14** | **Pergdp (RMB per capita)** | average GDP of the city |
| **15** | **Ctydoc (per thousand)** | Public and private doctor number of the city per thousand people |
| **16** | **Persave (RMB per capita)** | resident average bank saving |
| **17** | **Internet (per thousand)** | internet users of the city per thousand |
| **18** | **Bus (per 10 thousand)** | Public bus number per 10 thousand people |
| **19** | **Road (m^2^)** | City road square per capita |
| **20** | **lnpergdp** | ln(per_GPD) |
| **21** | **lnpersave** | ln(per_save) |
| **22** | **Ethirpeo2** | **(Sethirdpeo)^2^** |
| **23** | **Lnpergdp2** | (**lnpergdp)^2^** |

1. China CSEID of NSB of. China City Statistical Yearbook. Beijing: China Statistics Press; 2015.
